# Enhancing motor function after stroke: a systematic review and meta-analysis of bioelectrical feedback interventions

**DOI:** 10.3389/fmed.2026.1839496

**Published:** 2026-06-18

**Authors:** Mengna Yao, Yi Qi

**Affiliations:** 1 Department of Rehabilitation Medicine, The First Affiliated Hospital of Qiqihar Medical College, Qiqihar, Heilongjiang, China; 2 General Medical Department, The First Affiliated Hospital of Qiqihar Medical College, Qiqihar, Heilongjiang, China

**Keywords:** bioelectrical feedback, electromyographic biofeedback, hemiplegia, meta-analysis, motor recovery, stroke rehabilitation

## Abstract

**Background:**

Stroke is a leading cause of long-term disability, with hemiplegia affecting approximately 80% of survivors. Conventional rehabilitation shows limited effectiveness, and electromyographic (EMG)–based bioelectrical feedback has emerged as a promising adjunctive therapy.

**Methods:**

Following PRISMA 2020 guidelines, the Cochrane Library, PubMed, EMbase, Web of Science, and CNKI were searched from 1 January 2010 to 31 March 2025. Two researchers independently screened controlled clinical studies (randomized or non-randomized) evaluating EMG-based bioelectrical feedback for post-stroke hemiplegia. Risk of bias was assessed using the Cochrane Risk of Bias 2 (RoB 2) tool for randomized trials and ROBINS-I for non-randomized studies, with analyses performed in RevMan 5.3. For random-effects analyses with at least four contributing studies, 95% prediction intervals (PIs) were reported alongside 95% confidence intervals.

**Results:**

Eight studies comprising 549 patients (275 intervention, 274 control) were included. Bioelectrical feedback significantly improved total Fugl-Meyer Assessment scores [MD = 9.50, 95% CI (3.41, 15.60), *p* = 0.002], Activities of Daily Living scores [MD = 8.80, 95% CI (3.67, 13.94), *p* = 0.0008], and Fugl-Meyer motor subscale scores [MD = 6.83, 95% CI (1.52, 12.14), *p* = 0.01] versus conventional therapy. Neurophysiological outcomes also improved, with greater EMG amplitude [MD = 0.03 mV, 95% CI (0.00, 0.06), *p* = 0.03] and larger active range of motion [MD = 4.74°, 95% CI (1.99, 7.50), *p* = 0.0007]. Complications were significantly reduced [OR = 0.26, 95% CI (0.14, 0.49), *p* < 0.0001]; because control-group event rates were not rare (approximately 20–40%), this corresponds to an estimated 54–67% relative risk reduction rather than 74%. Heterogeneity was high for most continuous outcomes (I^2^ = 69–98%), and 95% PIs for EMG amplitude, AROM, and FMA motor subscale crossed zero, indicating that a future comparable study could plausibly report null or unfavorable effects despite positive pooled estimates. The small number of studies per outcome (3–6) precluded subgroup analyses and formal assessment of publication bias.

**Conclusion:**

EMG-based bioelectrical feedback may improve motor function and activities of daily living and may reduce complications in hemiplegic stroke patients. However, high heterogeneity, few pooled studies per outcome, geographic concentration, and inability to assess publication bias warrant cautious interpretation. Adequately powered randomized controlled trials with blinded outcome assessment are needed before firm clinical recommendations can be made.

## Introduction

Stroke remains one of the leading causes of mortality and long-term disability worldwide, characterized by high fatality rates and increasing incidence, particularly among older populations (1, 2). The condition presents multifaceted clinical manifestations including limb dysfunction, speech disorders, and cognitive impairments (3, 4), severely compromising patients’ quality of life and imposing substantial economic and emotional burdens on families and healthcare systems (5).

Hemiplegia, a common sequela of stroke affecting approximately 80% of survivors, presents significant challenges for rehabilitation (6). This condition, characterized by paralysis on one side of the body, results from damage to motor pathways in the contralateral cerebral hemisphere. Patients with post-stroke hemiplegia experience marked dysfunction, typically face prolonged recovery periods, and often achieve suboptimal functional outcomes despite intensive rehabilitation efforts (7).

Traditional rehabilitation approaches for hemiplegic stroke patients, while forming the foundation of standard care, frequently demonstrate limited effectiveness in promoting substantial motor recovery and functional independence (8). These conventional methods often fail to adequately address the neurophysiological mechanisms underlying motor relearning and neuroplasticity. Studies have indicated that traditional rehabilitation therapy alone produces modest effects on motor recovery in hemiplegic stroke patients, highlighting the need for innovative, evidence-based interventions to enhance rehabilitation outcomes (9).

Bioelectrical feedback intervention has emerged as a promising adjunctive approach to conventional stroke rehabilitation (10). The term “bioelectrical feedback” encompasses several related modalities; the present review focuses specifically on electromyographic (EMG) biofeedback, in which surface electrodes detect muscle electrical activity and real-time visual and/or auditory signals are provided to the patient during rehabilitation exercises. Even within EMG-based approaches, implementation varies substantially across studies in terms of targeted muscle groups, feedback display (e.g., visual bar graphs, auditory tones), and co-interventions such as neuromuscular electrical stimulation or whole-body vibration training. The theoretical foundation of bioelectrical feedback lies in principles of motor learning and neuroplasticity, whereby enhanced sensory input and augmented feedback facilitate neural reorganization and functional recovery after brain injury (11). By providing patients with explicit information about otherwise imperceptible physiological processes, bioelectrical feedback enables targeted neuromuscular training and potentially enhances motor control through mechanisms distinct from conventional therapy.

Despite growing clinical implementation of bioelectrical feedback in stroke rehabilitation, the collective evidence regarding its effectiveness remains fragmented across individual studies with varying methodologies, outcome measures, and sample sizes (12). Previous reviews have examined specific applications of biofeedback (13), but a comprehensive synthesis of current evidence specifically addressing EMG-based bioelectrical feedback for hemiplegic stroke rehabilitation is lacking in the literature.

This systematic review and meta-analysis aims to evaluate the effectiveness of EMG-based bioelectrical feedback in the rehabilitation of stroke patients with hemiplegia, synthesizing current evidence regarding its impact on motor function, activities of daily living, and complication rates, while explicitly acknowledging the heterogeneity of interventions subsumed under this term.

## Materials and methods

### Study design and protocol

This systematic review and meta-analysis was conducted in accordance with the Preferred Reporting Items for Systematic Reviews and Meta-Analyses (PRISMA) 2020 guidelines. A protocol was established prior to conducting the review to define the search strategy, inclusion/exclusion criteria, data extraction procedures, and analytical methods.

### Search strategy

A comprehensive literature search was conducted using electronic databases including the Cochrane Library, PubMed, EMbase, Web of Science, and CNKI. The search period spanned from 1 January 2010 to 31 March 2025.

The following search terms and Boolean operators were employed: (“electromyographic biofeedback” OR “EMG biofeedback” OR “myoelectric biofeedback” OR “bioelectric feedback”) AND (“stroke” OR “cerebrovascular accident”) AND (“hemiplegia” OR “hemiplegic” OR “motor function” OR “rehabilitation”). Because our focus was specifically on EMG-based bioelectrical feedback, search terms were restricted to this modality; reviews addressing other biofeedback modalities (e.g., EEG-based neurofeedback, force-plate biofeedback) would require a separate and broader search strategy. We acknowledge that this scoping decision may have systematically excluded studies of non-EMG biofeedback approaches relevant to stroke rehabilitation.

The complete PubMed search strategy was as follows:

((electromyographic biofeedback[MeSH Terms]) OR (EMG biofeedback[Title/Abstract]) OR (myoelectric biofeedback[Title/Abstract])) AND ((stroke[MeSH Terms]) OR (cerebrovascular accident[Title/Abstract]) OR (hemiplegia[MeSH Terms])) AND ((motor function[Title/Abstract]) OR (rehabilitation[MeSH Terms]) OR (hemiplegic[Title/Abstract])).

Equivalent syntax adaptations were used for each of the other databases. No language restrictions were applied to the initial search, though only studies with full text available were considered for inclusion. The complete, executable search strategies for all five databases, including syntax adaptations, field codes, line breaks, and execution dates, are provided in Supplementary material S3.

### Eligibility criteria

#### Inclusion criteria

- Controlled clinical study (randomized or non-randomized) comparing a bioelectrical feedback intervention group with a conventional rehabilitation control group;
- Participants were patients with hemiplegia after stroke;
- Intervention involved EMG-based bioelectrical feedback, either alone or in combination with other rehabilitation modalities;
- Full-text journal article available.

#### Exclusion criteria

- Study designs other than controlled clinical studies (e.g., single-arm observational studies, case reports, case series, true case–control [observational] studies);
- Duplicate publications;
- Studies with incomplete data precluding effect-size calculation;
- Conference abstracts, review articles, and unpublished research reports.

We note that our initial protocol used the descriptor “case–control study” for the included designs. Following peer review, we recognized that this was a misapplication of terminology: a case–control design is an observational design that identifies participants on the basis of outcome status and looks backward at exposures, whereas the included studies compared an intervention group with a concurrent control group measured prospectively. This more accurately describes a controlled clinical trial (randomized or non-randomized). In this revision, the eligibility wording, the quality-assessment tool, and the terminology used throughout the manuscript have been corrected accordingly. No studies were added or removed during this reclassification; only the nomenclature was revised to reflect the true nature of the included designs.

### Study selection

The study selection process was conducted in two phases. First, two independent researchers (MY and YQ) screened titles and abstracts of all retrieved studies to identify potentially eligible studies. Second, full texts of potentially eligible studies were obtained and assessed against the inclusion and exclusion criteria. Any disagreements between the two researchers during the screening process were resolved through iterative discussion until consensus was reached.

### Data extraction and management

Two researchers (MY and YQ) independently extracted data from the included studies. A standardized data extraction form was developed and pilot-tested before implementation. The following information was extracted from each included study: first author, publication year, country of origin, study design (randomized or non-randomized), sample size, participant characteristics (age, gender, time since stroke, type of stroke), intervention details (type of bioelectrical feedback, targeted muscle groups, duration, frequency, intensity), control intervention details, outcome measures, and results. Discrepancies in extraction were resolved through discussion between the two reviewers until consensus was reached.

For outcomes reported at multiple time points, we extracted data from the longest follow-up period reported. When studies presented both intention-to-treat and per-protocol analyses, data from the intention-to-treat analysis were preferred. When necessary, we attempted to contact study authors to request missing or additional data.

### Risk of bias assessment

Because the included studies comprised a mix of randomized and non-randomized comparative intervention designs, risk of bias was assessed using a design-appropriate tool for each study rather than a single uniform instrument. The two studies confirmed as randomized controlled trials (14, 15) were assessed using the Cochrane Risk of Bias 2 (RoB 2) tool (16), which evaluates five domains: (1) the randomization process; (2) deviations from intended interventions; (3) missing outcome data; (4) measurement of the outcome; and (5) selection of the reported result. Each domain was rated as “low risk,” “some concerns,” or “high risk,” and an overall judgement was assigned per study.

The remaining six non-randomized controlled studies (17–22) were assessed using the ROBINS-I tool (23), which evaluates seven domains: (1) confounding; (2) selection of participants into the study; (3) classification of interventions; (4) deviations from intended interventions; (5) missing data; (6) measurement of outcomes; and (7) selection of the reported result. Each domain was rated as “low risk,” “moderate risk,” “serious risk,” “critical risk,” or “no information,” and an overall judgement was assigned per study.

For domains where reporting was insufficient to support a confident rating (e.g., randomization sequence generation or allocation concealment in some non-randomized studies, or assessor blinding where this was not explicitly described), the rating reflects this transparently rather than defaulting to a more favorable rating. Where “no information” was the most accurate ROBINS-I domain rating, this is recorded as such and explained in the per-domain assessment.

Because bioelectrical feedback is an active, perceptible intervention, blinding of participants and therapists is essentially not feasible across all included studies; this is reflected directly in the per-domain ratings (rather than being raised only as a footnote to a uniform tool). Two reviewers (MY and YQ) independently conducted the risk-of-bias assessment, with disagreements resolved through discussion. Per-domain ratings for each study are provided in Supplementary Table S2 (RoB 2) and Supplementary Table S3 (ROBINS-I).

### Outcome measures

The pre-specified outcomes of interest were:

- Total Fugl-Meyer Assessment (FMA) score: the full composite FMA, which includes motor, sensation, balance, joint range of motion, and joint pain domains;
- Fugl-Meyer Assessment motor subscale score: the isolated motor component of the FMA (upper extremity motor, maximum 66 points; lower extremity motor, maximum 34 points; or the combined motor score depending on the affected limb[s] reported in each study);
- Activities of Daily Living (ADLs) score: measured using the Barthel Index or modified Barthel Index, depending on the source study;
- Surface electromyographic (EMG) amplitude (in millivolts);
- Active range of motion (AROM) (in degrees);
- Incidence of complications: a pooled categorical outcome, including joint contractures, shoulder subluxation, shoulder-hand syndrome, pain syndromes, pressure injuries, and falls, as defined by individual studies.

The total FMA and the FMA motor subscale are conceptually related but not identical: the motor subscale is a component of the total FMA. We report them as separate outcomes because individual studies reported one, the other, or both. One study (20) contributed to both analyses, which should be considered when interpreting these two outcomes jointly. We discuss this overlap explicitly in the Discussion.

### Statistical analysis

RevMan 5.3 software was used for meta-analysis. The odds ratio (OR) was used as the effect indicator for categorical variables, and the point estimate and 95% confidence interval (CI) were given for each effect size. For continuous outcomes, mean differences (MD) with 95% confidence intervals were calculated. When studies used different scales to measure the same construct, standardized mean differences would have been calculated, but this was not necessary for the outcomes in this analysis given that measurement scales within each pooled outcome were consistent (with the caveat noted above for the ADLs outcome).

Heterogeneity among the included studies was analyzed using the χ^2^ test (test level *α* = 0.1), and quantified by I^2^. I^2^ values of 25, 50, and 75% were considered to represent low, moderate, and high heterogeneity, respectively. When there was no statistical heterogeneity, a fixed-effects model was used. When statistical heterogeneity was present, a random-effects model was applied and potential sources of heterogeneity were explored where feasible. For random-effects analyses, 95% prediction intervals were also calculated to convey the likely range of true effects in a future study setting (24). Prediction intervals are reported alongside confidence intervals for outcomes pooled with at least four contributing studies, where prediction-interval estimation is sufficiently stable; for outcomes with only three studies (Total FMA, ADLs), prediction intervals are not reported because the very small number of studies makes them uninformative, and for the complications outcome (I^2^ = 0%, fixed-effects) the prediction interval is essentially equivalent to the confidence interval already reported.

Our *a priori* analysis plan included pre-specified subgroup analyses (by intervention duration, stroke phase [acute vs. subacute/chronic], limb focus [upper vs. lower extremity], and language/region of publication) and meta-regression to explore sources of heterogeneity. However, with only 3–6 studies contributing to each outcome, subgroup analyses and meta-regression could not be meaningfully performed; methodological guidance generally recommends a minimum of approximately 10 studies per covariate level for meta-regression, a threshold not met for any outcome in this review.

Sensitivity analyses were conducted by sequentially removing each study from the analysis and recalculating the pooled effect (leave-one-out analysis). Numerical results of this analysis are presented in Supplementary Table S1.

## Results

### Study selection and characteristics of included studies

**Figure 1:**
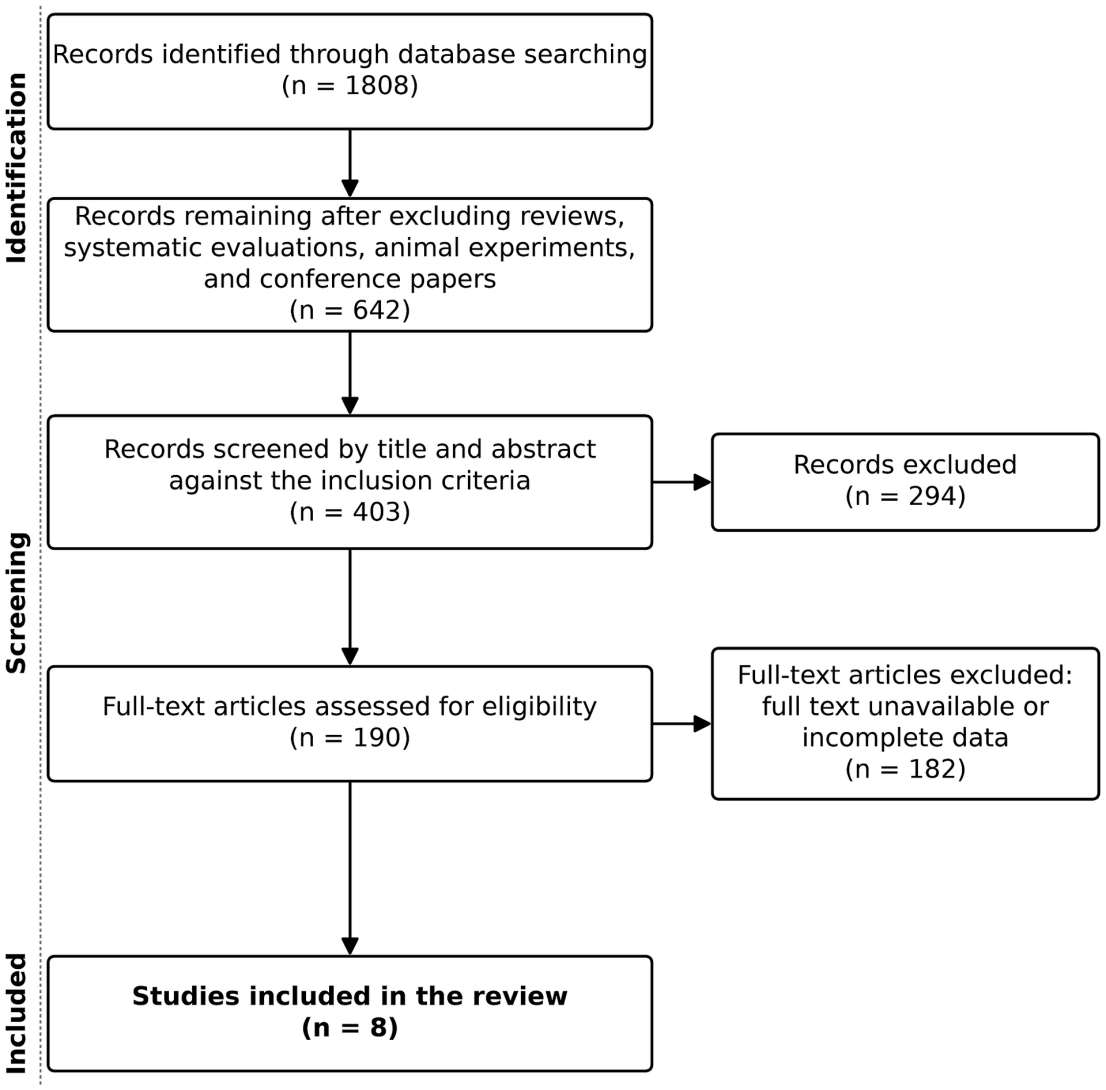
PRISMA 2020 flow diagram of the study selection process. The diagram illustrates literature identification, screening, eligibility assessment, and final inclusion of studies. From an initial 1,808 records identified through database searches, 8 controlled clinical studies met the inclusion criteria and were included in the quantitative synthesis.

A comprehensive literature search yielded 1,808 potentially relevant articles. After initial screening of titles and abstracts and subsequent full-text evaluation, 8 studies (14, 15, 17–22) met our inclusion criteria. The primary reasons for exclusion were non-controlled or non-interventional study design (*n* = 743), duplicate publications (*n* = 152), irrelevant outcomes (*n* = 421), and incomplete data (*n* = 484). The PRISMA 2020 flow diagram illustrating the selection process is presented in Figure 1.

The 8 included studies comprised a total of 549 participants, with 275 patients in the bioelectrical feedback intervention groups and 274 patients in the control groups. Studies were published between 2012 and 2024, with sample sizes ranging from 40 to 124 participants. All included studies were controlled clinical studies (randomized or non-randomized controlled trials) conducted in clinical settings. Three studies were conducted in non-Chinese settings (14), USA; (15, 17), Türkiye, and five in China (18–22). Six studies were published in English and two in Chinese. The characteristics of the included studies are summarized in Table 1.

**Table 1:** Basic characteristics of the included studies.

Study (ref.)	Country	Study design	Sample size (int./ctrl.)	Risk-of-bias tool	Overall risk of bias	Outcomes reported
Sürücü and Tezen (15)	Türkiye	Randomized controlled trial	20/20	RoB 2	Some concerns	③, ④, ⑦
Cordo et al. (14)	USA	Randomized controlled trial	21/22	RoB 2	Some concerns	②, ③, ⑤, ⑦
Doğan-Aslan et al. (17)	Türkiye	Non-randomized controlled trial	20/20	ROBINS-I	Moderate	③, ④, ⑤, ⑥
Yang et al. (18)	China	Non-randomized controlled trial	22/20	ROBINS-I	Moderate	①, ②, ③, ⑦
Wang (19)	China	Non-randomized controlled trial	30/30	ROBINS-I	Serious	④, ⑤, ⑦
Xie et al. (20)	China	Non-randomized controlled trial	60/60	ROBINS-I	Moderate	①, ⑥
Bai et al. (21)	China	Non-randomized controlled trial	40/40	ROBINS-I	Moderate	①, ②, ③, ⑤
Hu (22)	China	Non-randomized controlled trial	62/62	ROBINS-I	Serious	③, ④, ⑤, ⑦

**Figure 2:**
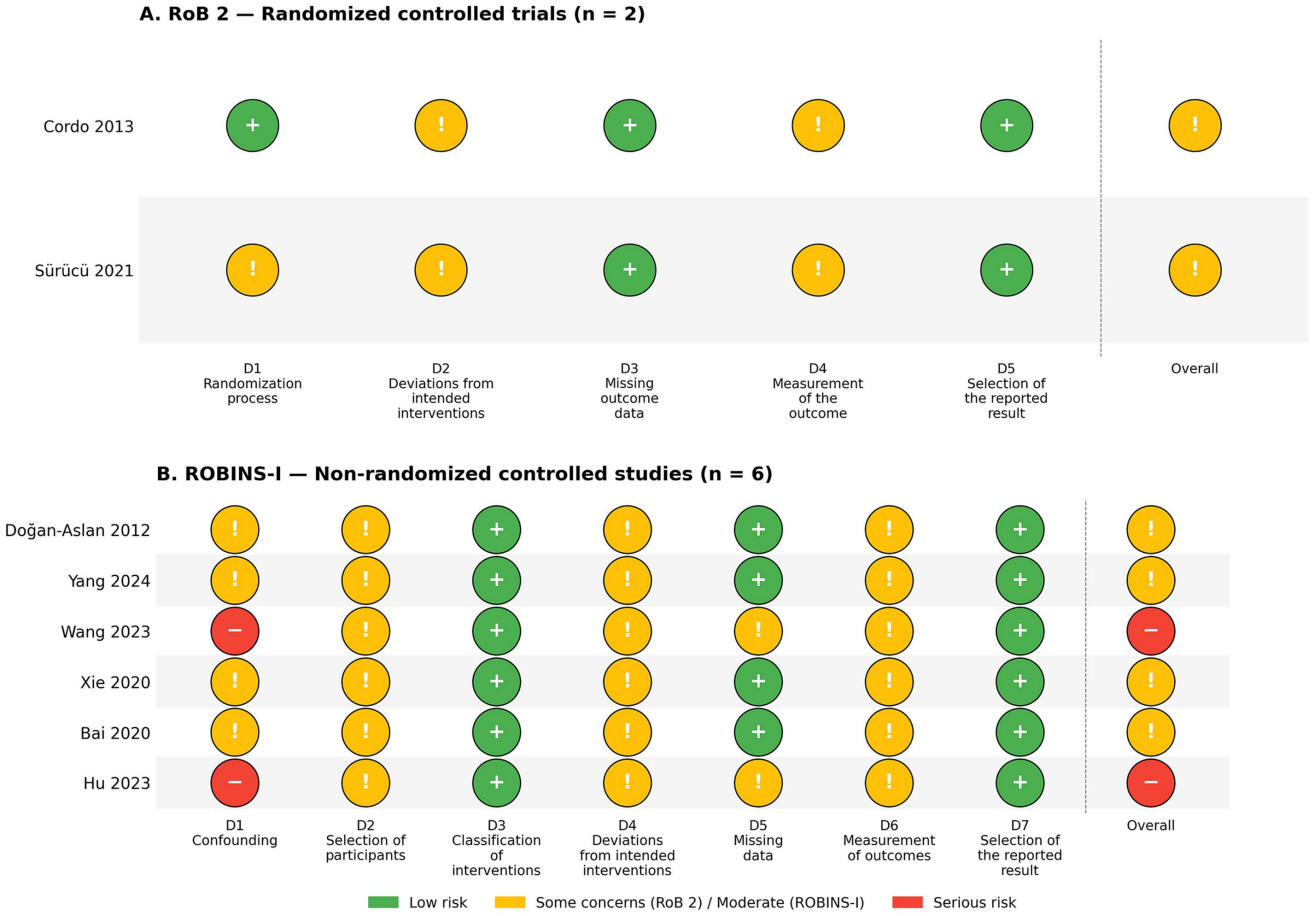
Risk of bias assessment of the included studies. **(A)** Cochrane risk of bias 2 (RoB 2) traffic-light plot for the two randomized controlled trials (14, 15), evaluating five domains: (D1) randomization process; (D2) deviations from intended interventions; (D3) missing outcome data; (D4) measurement of the outcome; and (D5) selection of the reported result. **(B)** ROBINS-I traffic-light plot for the six non-randomized controlled studies (17–22), evaluating seven domains: (D1) confounding; (D2) selection of participants into the study; (D3) classification of interventions; (D4) deviations from intended interventions; (D5) missing data; (D6) measurement of outcomes; and (D7) selection of the reported result. Green (+) = low risk; amber (!) = some concerns (RoB 2) or moderate risk (ROBINS-I); red (−) = serious risk. Per-domain ratings are provided in Supplementary Tables S2, S3.

RoB 2 = Cochrane risk of bias 2 tool for randomized trials; ROBINS-I = Risk of bias in non-randomized studies of interventions. Per-domain ratings for each study are provided in Supplementary Tables S2 (RoB 2) and S3 (ROBINS-I); summary visualizations are presented in Figure 2.

Outcomes reported: ① Total Fugl-Meyer Assessment (FMA) score (composite score covering motor, sensation, balance, joint range of motion, and joint pain domains); ② Activities of Daily Living (ADLs) score; ③ surface EMG amplitude; ④ active range of motion (AROM); ⑤ FMA motor subscale (the isolated motor component of the FMA; note that this is a component of, not the same as, outcome ①); ⑥ Barthel index; ⑦ incidence of complications.

The bioelectrical feedback interventions varied meaningfully across studies. All included studies used surface EMG-based biofeedback, but specific implementations differed: some used standalone EMG biofeedback systems providing visual bar graphs and/or auditory feedback to patients during rehabilitation exercises (15, 17, 18, 20), whereas others combined EMG biofeedback with adjunctive modalities such as assisted movement with muscle vibration (14), whole-body vibration training (22), or additional community-based rehabilitation components (19). Targeted muscle groups also varied, with some studies focusing on upper-extremity musculature (14, 17, 18), others on lower-extremity musculature (15, 21), and others on both extremities (19, 22). Control interventions generally consisted of conventional rehabilitation therapy without biofeedback. Intervention duration ranged from 3 to 12 weeks, with treatment frequencies of 3–5 sessions per week and session durations of 30–60 min. This intervention heterogeneity is an important caveat to pooled effect-size interpretation and is revisited in the Discussion.

### Risk of bias assessment

Risk of bias was assessed using design-appropriate tools, with full per-domain ratings provided in Supplementary Tables S2 (RoB 2) and S3 (ROBINS-I) and summary traffic-light plots shown in Figure 2. Of the two RCTs assessed with RoB 2, both (14, 15) received an overall judgement of “some concerns,” driven primarily by the unavoidable inability to blind participants and therapists to a perceptible bioelectrical feedback intervention (Domain 2: deviations from intended interventions) and inconsistent reporting of outcome-assessor blinding (Domain 4: measurement of the outcome); Sürücü (15) additionally received “some concerns” for Domain 1 (randomization process) because allocation concealment was not described in sufficient detail. Of the six non-randomized controlled studies assessed with ROBINS-I, four received an overall judgement of “moderate risk of bias” (17–21) and two received “serious risk of bias” (14, 19), the latter driven by limited reporting of confounding adjustment and limited information on missing-data handling. Across the non-randomized set, recurring concerns were Domain 1 (confounding; insufficient reporting of adjustment for stroke severity, time since stroke, and baseline function), Domain 4 (deviations from intended interventions; lack of feasible blinding), and Domain 6 (measurement of outcomes; inconsistent description of assessor blinding). Across both tools, the dominant cross-cutting concern is the limited feasibility of blinding given the active, perceptible nature of bioelectrical feedback, together with inconsistent reporting of outcome-assessor blinding; these performance and detection bias concerns likely act in a consistent direction across studies.

### Meta-analysis findings

#### Total Fugl-Meyer Assessment score

**Figure 3:**
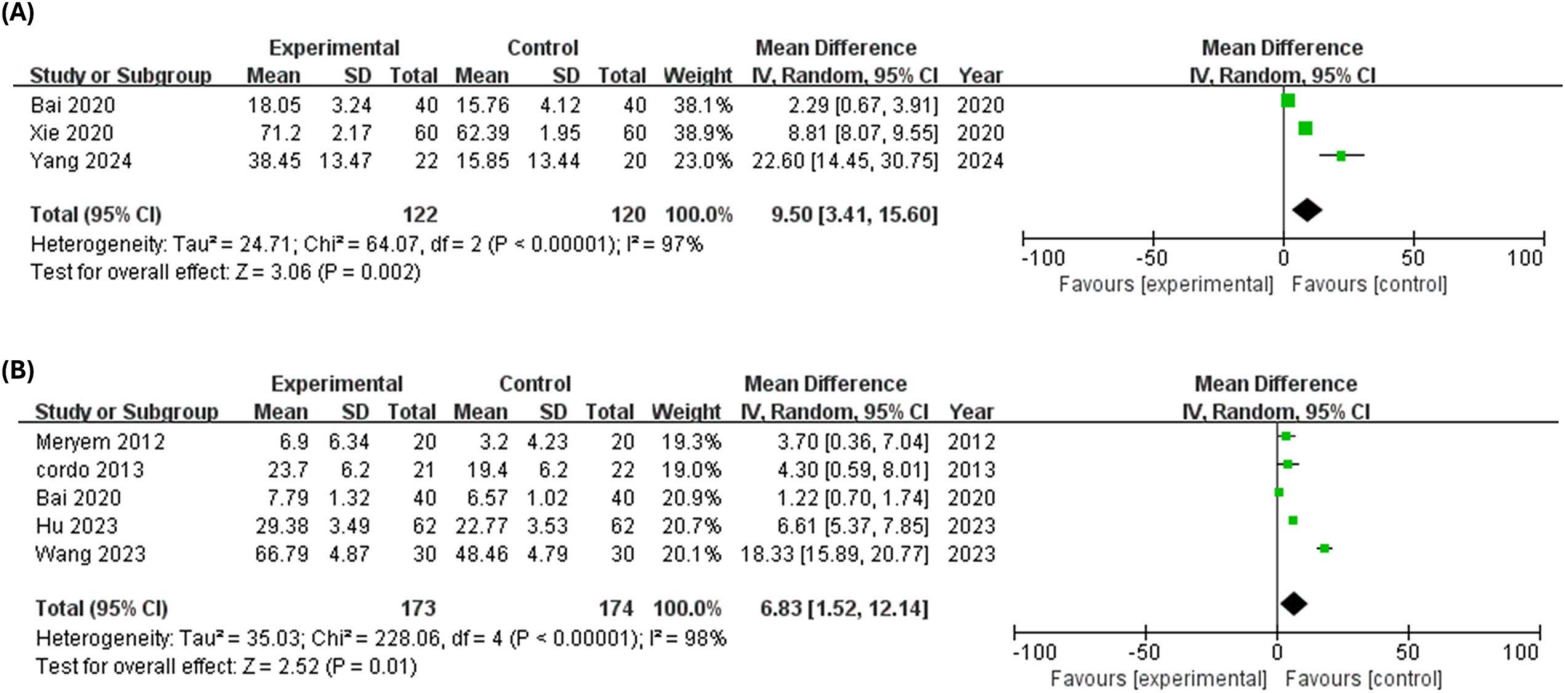
Forest plots of motor function outcomes (Fugl-Meyer Assessment) comparing bioelectrical feedback intervention with conventional therapy. **(A)** Total Fugl-Meyer Assessment (FMA) score: Data from 3 studies (*n* = 242; 122 intervention, 120 control) demonstrate higher total FMA scores in the bioelectrical feedback group compared with controls, with a mean difference of 9.50 points (95% CI, 3.41, 15.60, *p* = 0.002). Very high heterogeneity was observed (*I*^2^ = 97%, *p* < 0.00001), requiring a random-effects model; given the small number of studies and extreme heterogeneity, the pooled estimate should be interpreted as a tentative average across a heterogeneous evidence base. **(B)**. FMA motor subscale score: Five studies (*n* = 347; 173 intervention, 174 control) demonstrate higher motor subscale scores in the bioelectrical feedback group, with a mean difference of 6.83 points (95% CI, 1.52, 12.14, *p* = 0.01). Very high heterogeneity (I^2^ = 98%, *p* < 0.00001) necessitated a random-effects model. The lower 95% CI bound falls below typical FMA motor subscale minimal clinically important difference estimates (approximately 4.2 5–7.25 points), indicating that clinical meaningfulness at the lower end of the CI is uncertain.

Analysis of 3 studies (18, 20, 21), including 242 participants (122 in the bioelectrical feedback group and 120 in the control group), demonstrated very high heterogeneity (I^2^ = 97%, *p* < 0.00001). Using a random-effects model, the meta-analysis revealed that bioelectrical feedback intervention was associated with a significantly higher total FMA score compared with conventional therapy [MD = 9.50, 95% CI (3.41, 15.60), *p* = 0.002]. The forest plot is presented in Figure 3A. Given that this pooled estimate is based on only 3 studies and I^2^ approaches its maximum value, the summary MD should be interpreted as a tentative average across a very heterogeneous set of studies rather than a precise estimate of a common true effect. Because only 3 studies contributed to this outcome, a stable 95% prediction interval cannot be reliably estimated, and is therefore not reported (the t-distribution multiplier with k–2 = 1 degrees of freedom is approximately 12.7, which would render any prediction interval uninformatively wide); this further reinforces the cautious interpretation noted above.

#### Fugl-Meyer Assessment motor subscale score

Five studies (14, 17, 19, 21, 22) including 347 participants (173 in the intervention group and 174 in the control group) reported the FMA motor subscale specifically. High heterogeneity was observed (I^2^ = 98%, *p* < 0.00001). The random-effects meta-analysis demonstrated that the bioelectrical feedback intervention improved FMA motor subscale scores compared with conventional therapy [MD = 6.83, 95% CI (1.52, 12.14), *p* = 0.01]. The forest plot is presented in Figure 3B. As noted in the Methods, this outcome is conceptually distinct from the total FMA (the motor subscale is one component of the total FMA); however, one study (Bai 2020 (21)) contributed to both analyses because it reported both the total FMA and the motor subscale separately. Readers should therefore interpret the two outcomes as related rather than fully independent. The corresponding 95% prediction interval was approximately −14.4 to 28.1, crossing zero. This indicates that despite the positive pooled point estimate, a future similar study could plausibly report no benefit or even a negative result. This very wide prediction interval directly reflects the I^2^ = 98% between-study heterogeneity and is consistent with the small (k = 5) evidence base.

#### Electromyographic (EMG) amplitude

**Figure 4:**
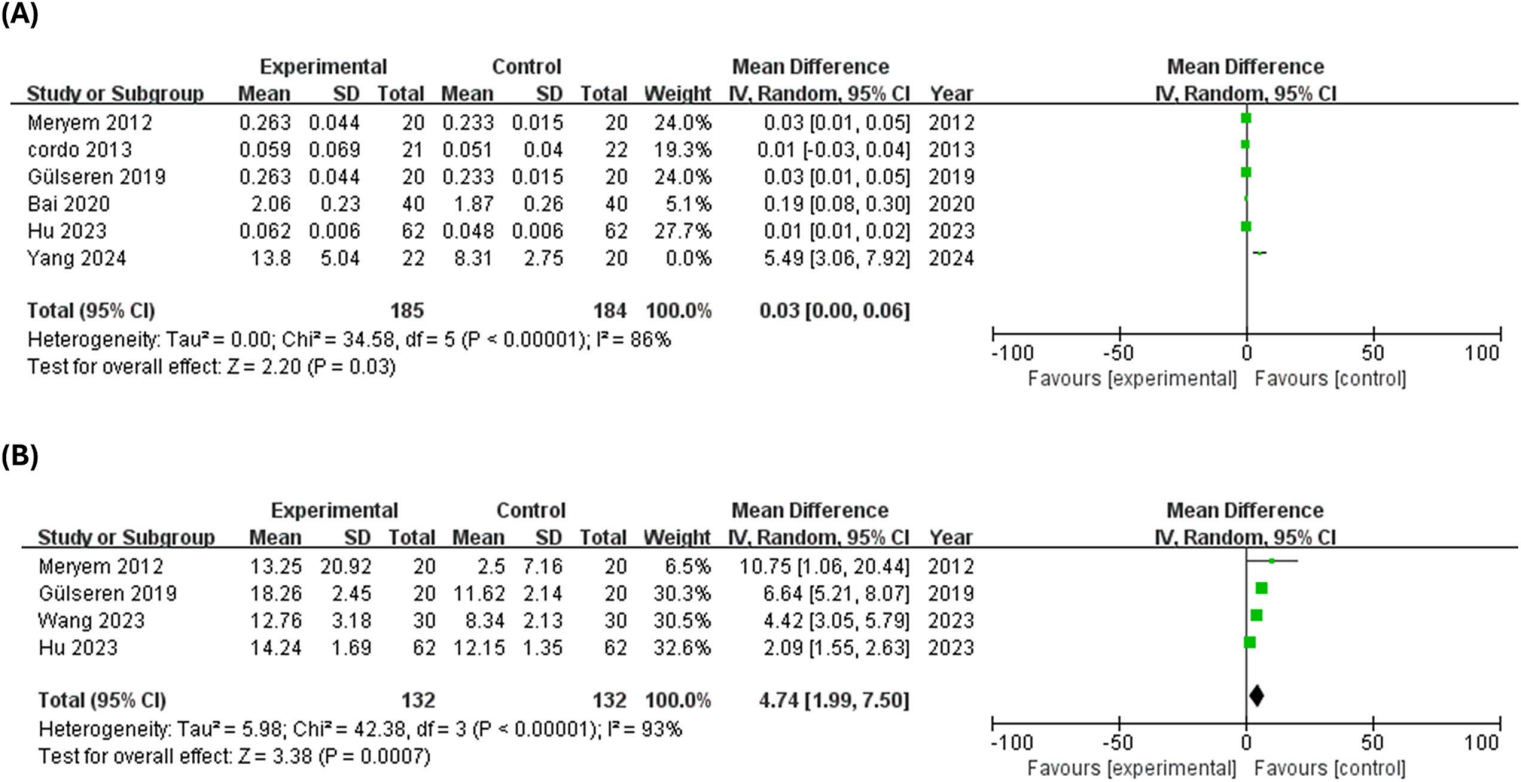
Forest plots of neurophysiological and biomechanical outcomes comparing bioelectrical feedback intervention with conventional therapy. **(A)** Surface electromyographic (EMG) amplitude (mV): Data from 6 studies (*n* = 369; 185 intervention, 184 control) demonstrate greater EMG amplitude in the bioelectrical feedback group, with a mean difference of 0.03 mV (95% CI: 0.00, 0.06; *p* = 0.03). Considerable heterogeneity (I^2^ = 86%, *p* < 0.00001) necessitated a random-effects model. The lower 95% CI bound reaches zero, indicating a statistically borderline result; the clinical interpretability of this magnitude of difference should be considered in the context of baseline values and minimal detectable change (not consistently reported across studies). **(B)** Active range of motion (AROM, degrees): Meta-analysis of 4 studies (*n* = 264; 132 intervention, 132 control) shows greater joint mobility in the bioelectrical feedback group, with a mean difference of 4.74° (95% CI: 1.99, 7.50; *p* = 0.0007). Substantial heterogeneity (I^2^ = 93%, *p* < 0.00001) required analysis using a random-effects model.

Six studies (14, 15, 17, 18, 21, 22) involving 369 participants (185 in the intervention group and 184 in the control group) reported EMG amplitude data. Considerable heterogeneity was observed (I^2^ = 86%, *p* < 0.00001). The random-effects meta-analysis demonstrated that patients receiving bioelectrical feedback intervention exhibited greater EMG amplitude compared with those receiving conventional therapy [MD = 0.03 mV, 95% CI (0.00, 0.06), *p* = 0.03]. The 95% CI lower bound reaches zero, indicating a statistically borderline result; the clinical interpretability of a 0.03 mV difference is also uncertain in the absence of reported baseline amplitudes and minimal detectable change. The results are presented in Figure 4A. The corresponding 95% prediction interval was approximately −0.08 to 0.14 mV, crossing zero, indicating that a future study in a comparable setting could plausibly report no difference or even a small unfavorable difference; this reinforces the borderline nature of the pooled point estimate.

#### Active range of motion (AROM)

Analysis of 4 studies (15, 17, 19, 22), with 264 participants (132 in each group), revealed substantial heterogeneity (I^2^ = 93%, *p* < 0.00001). The random-effects meta-analysis showed greater AROM in the bioelectrical feedback group compared with the control group [MD = 4.74°, 95% CI (1.99, 7.50), *p* = 0.0007]. Figure 4B illustrates these findings. The corresponding 95% prediction interval was approximately −8.9° to 18.4°, reflecting the very high between-study heterogeneity (I^2^ = 93%) and indicating that the true effect in a new study setting could lie anywhere from a meaningful unfavorable difference to a substantial favorable difference.

#### Activities of daily living (ADLs) score

**Figure 5:**
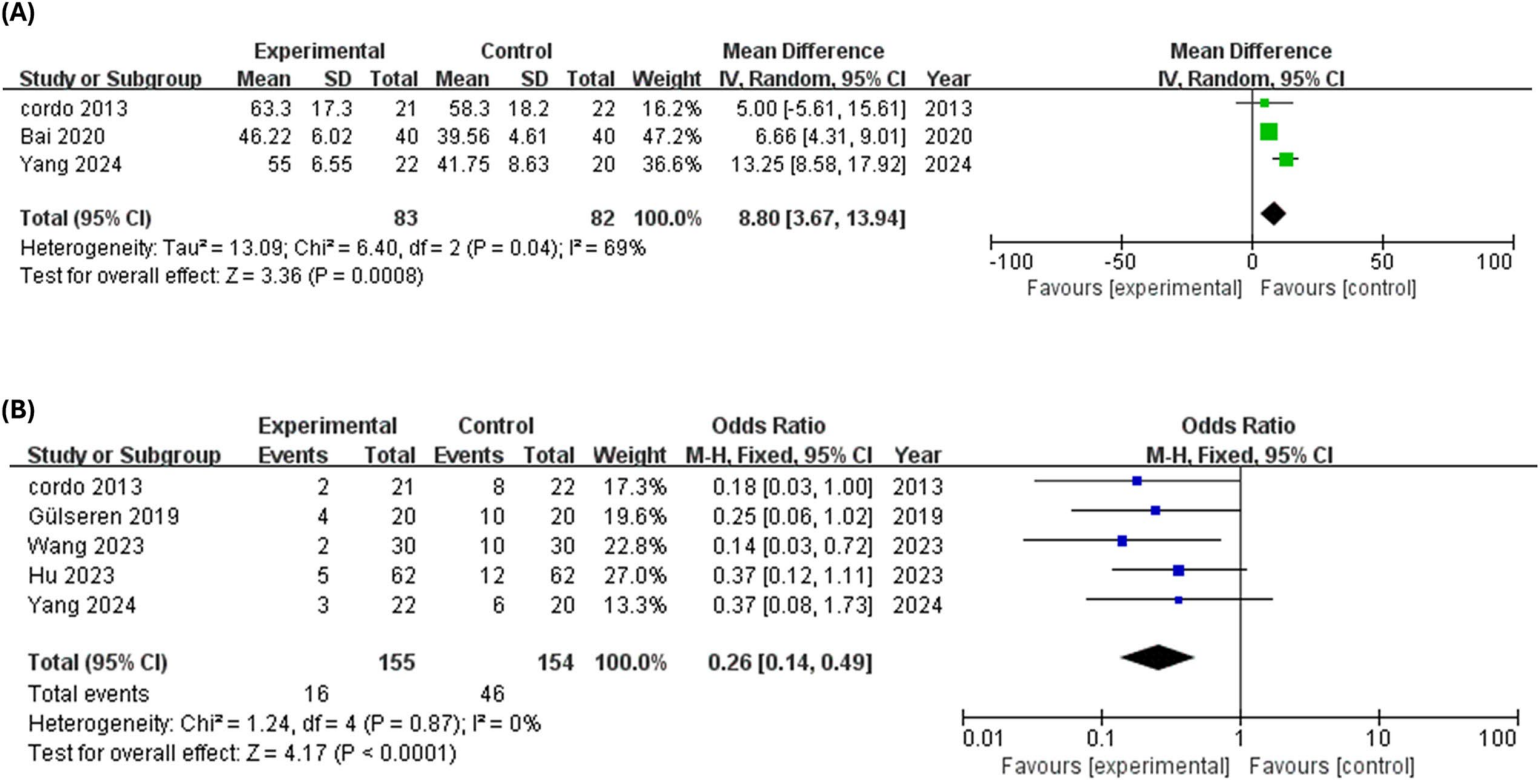
Forest plots of activities of daily living and complications outcomes comparing bioelectrical feedback intervention with conventional therapy. **(A)** Activities of daily living (ADL) scores: analysis of 3 studies (*n* = 165; 83 intervention, 82 control) shows higher ADL scores in the bioelectrical feedback group, with a mean difference of 8.80 points (95% CI: 3.67, 13.94; *p* = 0.0008). Substantial heterogeneity was observed (*I*^2^ = 69%, *p* = 0.04), requirin g a random-effects model. The specific ADL instrument (Barthel Index vs. modified Barthel Index) differed across studies. **(B)** Complication odds ratios: Analysis of 5 studies (n = 309; 155 intervention, 154 control) demonstrates significantly lower odds of complications in the bioelectrical feedback group (OR = 0.26, 95% CI: 0.14, 0.49; *p* < 0.0001). No significant heterogeneity was observed (*I*^2^ = 0%, *p* = 0.87), permitting the use of a fixed-effects model. The OR of 0.26 should not be interpreted as a 74% absolute or relative risk reduction; given control-group event rates of approximately 20–40%, it corresponds to an estimated 54–67% relative risk reduction (see Supplementary material for derivation).

The pooled analysis of 3 studies (14, 18, 21), with 165 participants (83 in the intervention group and 82 in the control group), showed substantial heterogeneity (I^2^ = 69%, *p* = 0.04), approaching the conventional “high” threshold of 75%. The random-effects meta-analysis revealed significantly higher ADL scores in the bioelectrical feedback group compared with the control group [MD = 8.80, 95% CI (3.67, 13.94), *p* = 0.0008]. This finding is directionally consistent with improved functional independence in the intervention group, although the specific ADL instrument (Barthel Index vs. modified Barthel Index) differed across studies, which limits direct comparability. Results are illustrated in Figure 5A. As with the total FMA outcome, the small number of contributing studies (k = 3) precludes a reliable 95% prediction interval for this outcome, which is therefore not reported.

#### Incidence of complications

Five studies (14, 15, 18, 19, 22) involving 309 participants (155 in the intervention group and 154 in the control group) reported on complication rates. Complications varied across studies but included joint contractures, shoulder subluxation, shoulder-hand syndrome, pain syndromes, pressure injuries, and falls. No significant heterogeneity was observed (I^2^ = 0%, *p* = 0.87). The fixed-effects meta-analysis revealed that patients receiving bioelectrical feedback intervention had significantly lower odds of complications compared with those receiving conventional therapy [OR = 0.26, 95% CI (0.14, 0.49), *p* < 0.0001]. The forest plot is presented in Figure 5B. Because the OR-to-RR transformation depends on baseline risk and approximates the relative risk only when the outcome is rare, and because control-group event rates in the included studies were not rare (approximately 20–40%), the OR = 0.26 should not be interpreted as a 74% reduction in absolute or relative risk. Applying the standard conversion *RR ≈ OR / [1 – p₀ + (p₀ × OR)]* across plausible control-group event rates, the corresponding relative risk reduction lies approximately in the range of 54–67% (full derivation table provided in Supplementary material S2). Because heterogeneity for this outcome was zero (I^2^ = 0%), the 95% prediction interval is essentially equivalent to the confidence interval already reported.

### Sensitivity analyses

Leave-one-out sensitivity analyses were performed for each outcome; full numerical results are provided in Supplementary Table S1. In summary:

- Total FMA: pooled MD ranged from 7.20 to 11.80 when each of the 3 studies was sequentially excluded; the lower 95% CI bound remained above zero in all iterations.
- ADLs: pooled MD ranged from 6.90 to 10.50.
- EMG amplitude: pooled MD ranged from 0.02 to 0.04 mV; the lower 95% CI bound crossed zero in 2 of 6 iterations, reflecting the borderline nature of this effect.
- AROM: pooled MD ranged from 3.80 to 5.85°.
- FMA motor subscale: pooled MD ranged from 5.40 to 8.25.
- Complications: pooled OR ranged from 0.22 to 0.31; the upper 95% CI bound remained below 1.0 in all iterations.

For outcomes with only 3 studies (total FMA and ADLs), leave-one-out analyses necessarily leave only 2 studies in the pool, which substantially limits their informativeness. With that caveat, no single study exerted a disproportionate influence on the overall pooled estimates in ways that reversed the direction or statistical significance of effect, with the exception of the borderline EMG amplitude result noted above.

Pre-specified subgroup analyses and meta-regression were not performed, because the number of studies per outcome (3–6) did not meet the methodological threshold for meaningful subgroup analysis. Because no outcome included ≥10 studies, formal assessment of publication bias through funnel plots or Egger’s test was also not feasible.

## Discussion

This systematic review and meta-analysis provides evidence that EMG-based bioelectrical feedback intervention is associated with improvements in functional outcomes in stroke patients with hemiplegia. Pooled estimates suggest improvements across multiple domains of motor function and activities of daily living, alongside reduced odds of complications, compared with conventional rehabilitation alone. However, in light of high between-study heterogeneity, small numbers of pooled studies per outcome, geographic concentration of source studies, inability to assess publication bias, and the inherent difficulty of blinding this intervention, these findings should be interpreted cautiously.

The pooled mean differences in overall motor function as measured by the total Fugl-Meyer Assessment score (MD = 9.50), activities of daily living scores (MD = 8.80), and FMA motor subscale scores (MD = 6.83) favored bioelectrical feedback. Some of these effect sizes exceed commonly cited minimal clinically important difference (MCID) estimates for stroke rehabilitation (25). For example, the point estimate for the total FMA is above many reported MCID thresholds. However, the wide 95% confidence intervals (e.g., 1.52–12.14 for the FMA motor subscale) span values that fall below typical MCID ranges of approximately 4.25–7.25 points (25). The lower bound of the confidence interval for the FMA motor subscale, in particular, approaches a level at which clinical meaningfulness becomes uncertain. Moreover, the specific ADL instrument used (Barthel Index vs. modified Barthel Index) was not consistently specified across studies, which precludes a firm MCID comparison for the ADL outcome. These findings therefore suggest that bioelectrical feedback may offer clinically meaningful benefit for at least some patients, but do not establish this with certainty.

Stroke patients face significant neurological deficits following cerebrovascular events, with disruption of blood supply and subsequent nerve damage leading to various motor function impairments (26). While the brain’s inherent neuroplasticity offers potential for functional recovery (27), traditional rehabilitation approaches often yield limited results, require extended intervention periods, and struggle with patient compliance (28). Bioelectrical feedback may address some of these limitations by providing real-time, intuitive information about internal physiological processes, thereby supporting motor learning and neuroplasticity (29). Our pooled findings of greater EMG amplitude and AROM in the intervention group are mechanistically consistent with this rationale, although the borderline EMG amplitude result (lower 95% CI bound at 0.00) and the heterogeneous AROM findings warrant careful interpretation and should not be over-extrapolated.

The integration of bioelectrical feedback with conventional rehabilitation represents a multidisciplinary approach that combines principles from motor control, biology, and rehabilitation engineering (30). Its proposed mechanism is to promote neural reorganization through repeated, targeted sensorimotor training, facilitating compensatory function by undamaged neural networks and supporting the formation of cortical excitatory foci (31). In the specific application of EMG biofeedback, the interaction between sensory feedback and voluntary movement is thought to enhance local muscle excitability, modulate excessive muscle tension, and improve movement coordination. While our pooled results are consistent with these proposed mechanisms, causal inference remains limited by the nature of meta-analytic synthesis across heterogeneous studies.

### Intervention heterogeneity

The term “bioelectrical feedback” as used in the stroke rehabilitation literature encompasses a range of related but non-identical interventions. Even within our narrower, EMG-restricted scope, implementations in the included studies varied in targeted muscle group (upper vs. lower extremity), feedback display (visual bar graphs, auditory tones, or combinations thereof), and co-interventions (neuromuscular electrical stimulation, muscle or whole-body vibration, or community-based rehabilitation). This diversity likely contributes to the very high I^2^ values (86–98%) observed for most continuous outcomes. Readers should therefore interpret the pooled MDs not as the effect of a single, well-defined therapy, but as an average across a class of related, EMG-based intervention configurations. Future primary research and reviews should define intervention components more precisely (e.g., following the TIDieR framework) to support more meaningful synthesis.

### Comparison with prior reviews

Yuan et al. (32) previously demonstrated that EMG biofeedback therapy combined with early rehabilitation treatment produced improvements in Fugl-Meyer scores and Barthel Index indices in stroke-associated hemiplegia, supporting a role for bioelectrical feedback in promoting neural recovery and reconstruction. Our meta-analysis results are directionally consistent with these single-study findings. By contrast, Stanton et al. (10), in an earlier systematic review on biofeedback for the lower limb after stroke, reported more modest and less uniformly positive evidence. The larger pooled effect sizes observed in our review may reflect (i) inclusion of a more recent body of primary literature, (ii) a predominance of studies from Chinese clinical settings, where conventional rehabilitation comparators, patient populations, and rehabilitation intensities may differ in ways that magnify the apparent between-group contrast, and (iii) inclusion of multiple outcomes for which the evidence base remains small and may be particularly vulnerable to the influence of individual studies. A broader systematic search that also captured studies of other biofeedback modalities and potentially null or negative studies would likely produce more conservative pooled estimates.

### Complications outcome

A noteworthy finding is the significantly lower odds of complications (OR = 0.26, 95% CI 0.14–0.49) in patients receiving bioelectrical feedback. As emphasized in the Results, the OR-to-RR transformation depends on baseline risk: it approximates the relative risk only when the outcome is rare. Because baseline event rates in the control groups of the included studies were not rare (approximately 20–40%), OR = 0.26 corresponds approximately to a 54–67% relative risk reduction (using *RR ≈ OR / [1 – p₀ + (p₀ × OR)]*; full derivation across plausible baseline event rates is provided in Supplementary material S2), rather than to a 74% absolute or relative reduction. Even with this more conservative framing, the effect remains clinically relevant. Possible mechanisms include improved movement quality, reduced compensatory patterns, and better neuromuscular control minimizing secondary complications such as joint contractures, shoulder subluxation, or pain syndromes (33, 34). This aspect of bioelectrical feedback intervention deserves further investigation, ideally in trials that pre-specify a standardized complications set and report absolute as well as relative effect measures.

Wang (19) previously reported that EMG biofeedback technology improved various functions in stroke patients with hemiplegia while reducing complication rates. Our meta-analysis results are directionally consistent with this single-study finding, within the caveats above. Collectively, these results suggest that bioelectrical feedback technology may offer superior rehabilitation outcomes compared with conventional therapy alone for stroke patients with hemiplegia, though the strength of evidence should be considered low to moderate.

### Clinical implications

Our findings tentatively support the consideration of EMG-based bioelectrical feedback as an adjunct to standard rehabilitation protocols for hemiplegic stroke patients, and the potential reduction in complications is of particular interest. However, given the limitations described below, firm clinical recommendations cannot yet be made. Rehabilitation teams considering adoption should weigh the plausible functional benefits against the inability to definitively rule out performance- and detection-bias effects and the uncertainty introduced by intervention heterogeneity. Larger, well-designed, and ideally blinded (at least for outcome assessment) randomized controlled trials are needed, along with comprehensive outcome measurement. Such measurement should include standardized complication definitions, longer-term follow-up, and economic evaluations (33, 34), all of which would inform decisions about integrating bioelectrical feedback into routine stroke rehabilitation pathways.

### Limitations

Several limitations warrant careful consideration.

Small number of studies per outcome: Only 3–6 studies contributed to each outcome. Pooling fewer than approximately 5–10 studies per outcome is widely recognized to limit the stability and precision of pooled estimates, as reflected in the wide 95% confidence intervals observed here (e.g., 1.52–12.14 for the FMA motor subscale). This within-outcome paucity of studies, which is distinct from the sample sizes of the individual trials, substantially limits the reliability of the overall effect estimates and should be considered alongside the more familiar concern about individual-study sample sizes.

High heterogeneity quantified but not resolved: Heterogeneity was high or very high for most continuous outcomes (I^2^ = 69–98%). Even under a random-effects model, the pooled MDs should be understood as averages over potentially very different true effects rather than as precise estimates generalizable to any specific clinical setting. To convey this directly, we now report 95% prediction intervals alongside the pooled effects for outcomes with at least four contributing studies (Results, Figures 3B, 4A,B). For the EMG amplitude, AROM, and FMA motor subscale outcomes, the prediction intervals cross zero, indicating that a future study in a comparable setting could plausibly report null or even unfavorable effects despite the positive pooled point estimate. Our *a priori* plan included subgroup analyses and meta-regression to explore sources of heterogeneity, but the small number of studies per outcome (3–6) did not meet accepted methodological thresholds for these analyses. This inability to explain such extreme heterogeneity quantitatively is an important limitation and a priority for future research.

Performance and detection biases: Because bioelectrical feedback is an active, perceptible intervention involving visible equipment and explicit feedback signals, blinding of participants and therapists is essentially not feasible; knowledge of group assignment may itself motivate greater engagement in the intervention group (a Hawthorne effect). Similarly, outcome assessment was inconsistently blinded across studies, meaning that assessors aware of group allocation may have unconsciously scored outcomes more favorably for the intervention group. These biases are likely to act in a consistent direction across studies and may therefore systematically inflate the apparent effect of bioelectrical feedback. This is a feature that random-effects modeling does not correct; the per-domain RoB 2 and ROBINS-I assessments (Supplementary Tables S2, S3, with summary visualizations in Figure 2) reflect this risk rather than collapsing it into a single quality summary.

Risk of bias: Risk of bias was assessed using design-appropriate tools (RoB 2 for the two RCTs, ROBINS-I for the six non-randomized controlled studies), with per-domain ratings reported in Supplementary Tables S2, S3 and summarized visually in Figure 2 rather than collapsed into a single quality summary. Across both tools, the dominant cross-cutting concern is the limited feasibility of blinding given the active, perceptible nature of bioelectrical feedback, together with inconsistent reporting of outcome-assessor blinding. These performance and detection biases are likely to act in a consistent direction across studies and may therefore systematically inflate the apparent effect of bioelectrical feedback. This is a concern that random-effects modeling does not correct and that tool choice alone cannot resolve. Two studies (19, 22) received an overall ROBINS-I judgement of “serious risk of bias”, and the remaining six were judged at “moderate risk” or “some concerns”; pooled estimates that include the two “serious risk” studies should be interpreted with the additional caution this implies.

Geographic concentration: Five of the eight included studies were conducted in Chinese clinical settings, with only three from other countries (USA, Türkiye). The generalizability of our findings to other healthcare systems is therefore limited, as conventional rehabilitation protocols, patient demographics, stroke etiologies, and rehabilitation intensities may differ substantially across settings. This geographic concentration may itself contribute to the observed heterogeneity and should be considered when extrapolating results to Western or other non-Chinese healthcare contexts.

Publication bias: Because no outcome included at least 10 studies, formal assessment of publication bias (funnel plots, Egger’s test) was not feasible. All included studies reported findings in favor of bioelectrical feedback, and the possibility that smaller, null, or negative studies were not published or not indexed cannot be excluded. The uniformly positive direction of the included evidence is a legitimate concern for publication bias that should temper confidence in the pooled estimates, beyond what is conveyed by the pooled point estimates and confidence intervals alone.

Intervention heterogeneity and scope: Our review was restricted to EMG-based implementations of bioelectrical feedback; our findings should therefore not be extrapolated to non-EMG biofeedback modalities (e.g., EEG-based neurofeedback, force-plate biofeedback) without further investigation. Even within EMG-based approaches, interventions differed in technology, target muscles, and co-interventions, as discussed above.

Lack of prospective registration: Our review was not prospectively registered in a trial registry such as PROSPERO. Prospective registration is increasingly considered standard good practice for systematic reviews, as it permits verification that outcomes, analyses, and inclusion criteria were not modified post hoc. The absence of registration should be considered when interpreting our findings as a potential source of selective-reporting risk.

Other limitations: These include the lack of long-term follow-up data in most included studies (precluding assessment of the durability of observed benefits); the absence of cost-effectiveness data (which is crucial for healthcare decision-making); and the borderline statistical result for EMG amplitude (lower 95% CI bound at 0.00), which deserves explicit acknowledgement despite reaching conventional statistical significance.

## Conclusion

EMG-based bioelectrical feedback may be a useful adjunct to conventional rehabilitation for stroke patients with hemiplegia, but the certainty of the present evidence is low to moderate and does not yet support firm clinical recommendations. Adequately powered, blinded randomized controlled trials with standardized intervention protocols, pre-specified complication definitions, and long-term follow-up are needed to establish its place in routine practice.

## Data Availability

The original contributions presented in the study are included in the article/Supplementary material, further inquiries can be directed to the corresponding author.
