# Supplementary material for: Enhancing motor function after stroke: a systematic review and meta-analysis of bioelectrical feedback interventions
Source: Front Med (Lausanne). 2026 Jun 18;13:1839496. doi: 10.3389/fmed.2026.1839496 (PMC13322862; doi:10.3389/fmed.2026.1839496)
Supplement: Supplementary file 1 [file Table_1.docx]

**Supplementary Table S1. Leave-one-out sensitivity analysis for each meta-analytic outcome.**

| Outcome | N studies | Pooled estimate (main analysis) | Leave-one-out range (pooled effect) | Direction of effect retained in all iterations? | Statistical significance retained in all iterations? |
| --- | --- | --- | --- | --- | --- |
| Total FMA score | 3 | MD=9.50 (3.41, 15.60) | MD range: 7.20 to 11.80 | Yes | Yes |
| ADLs score | 3 | MD=8.80 (3.67, 13.94) | MD range: 6.90 to 10.50 | Yes | Yes |
| EMG amplitude (mV) | 6 | MD=0.03 (0.00, 0.06) | MD range: 0.02 to 0.04 | Yes | **No** – lower 95% CI bound crossed zero in 2 of 6 iterations |
| AROM (degrees) | 4 | MD=4.74 (1.99, 7.50) | MD range: 3.80 to 5.85 | Yes | Yes |
| FMA motor subscale | 5 | MD=6.83 (1.52, 12.14) | MD range: 5.40 to 8.25 | Yes | Yes |
| Complications | 5 | OR=0.26 (0.14, 0.49) | OR range: 0.22 to 0.31 | Yes | Yes |

*Notes:* For outcomes with only 3 studies (total FMA, ADLs), leave-one-out analyses result in pooled estimates based on only 2 remaining studies, which is widely considered too few for stable meta-analytic pooling; results for these outcomes should be interpreted with particular caution.

FMA = Fugl-Meyer Assessment; ADLs = Activities of Daily Living; EMG = Electromyographic; AROM = Active Range of Motion; MD = Mean Difference; OR = Odds Ratio; CI = Confidence Interval.

**Supplementary Note S1. Rationale for not performing subgroup and meta-regression analyses.**

The original protocol for this systematic review included planned subgroup analyses and meta-regression to explore sources of between-study heterogeneity, stratified by the following covariates:

- Intervention duration (≤6 weeks vs. >6 weeks);
- Stroke phase at study entry (acute/subacute vs. chronic);
- Limb focus (upper extremity vs. lower extremity);
- Language/region of publication (Chinese vs. non-Chinese).

In the completed review, however, only 3–6 studies contributed to each outcome. Methodological guidance (e.g., Cochrane Handbook for Systematic Reviews of Interventions, section 10.11.5.1) generally recommends a minimum of approximately 10 studies per covariate level for meta-regression, with even larger numbers preferred for stable subgroup comparisons. For every outcome in our review, the available evidence base falls well below this threshold: performing subgroup analyses with 1–3 studies per subgroup would produce estimates with very wide confidence intervals and a high risk of false-positive or false-negative subgroup findings. For transparency, we have explicitly documented both the planned subgroup structure and the reason these analyses were not performed, rather than reporting underpowered subgroup estimates.

This limitation is a priority for future research, which should aim to pool a larger set of primary studies (either by extending the search window, relaxing eligibility, or waiting for additional primary evidence) before attempting quantitative exploration of heterogeneity.

**Supplementary Note S2. Conversion between odds ratio and relative risk for the complications outcome.**

The pooled odds ratio for complications was OR=0.26 (95% CI: 0.14, 0.49). Because the odds ratio approximates the relative risk only when the outcome is rare (roughly <10%), and because control-group complication rates in the included studies were generally in the range of approximately 20–40%, the OR should not be interpreted as directly equivalent to a 74% risk reduction.

An approximate conversion using the formula RR ≈ OR / [1 – p₀ + (p₀ × OR)], where p₀ is the baseline control-group event rate:

| Baseline control event rate (p₀) | Approximate RR | Approximate relative risk reduction |
| --- | --- | --- |
| 20% | 0.30 | 70% |
| 25% | 0.33 | 67% |
| 30% | 0.37 | 63% |
| 35% | 0.41 | 59% |
| 40% | 0.46 | 54% |

Based on this conversion, the relative risk reduction implied by the pooled odds ratio most plausibly lies in the range of **54–67%** across the event-rate scenarios observed in the included studies, rather than the 74% reduction that would be inferred by directly equating OR to RR.

**Supplementary Table S2. Per-domain Cochrane Risk of Bias 2 (RoB 2) ratings for the two randomized controlled trials.**

| **Study (Ref.)** | **D1: Randomization process** | **D2: Deviations from intended interventions** | **D3: Missing outcome data** | **D4: Measurement of the outcome** | **D5: Selection of the reported result** | **Overall judgement** |
| --- | --- | --- | --- | --- | --- | --- |
| Cordo 2013 (15) | Low | Some concerns (no participant/therapist blinding feasible) | Low | Some concerns (assessor blinding not explicitly described) | Low | Some concerns |
| Sürücü 2021 (14) | Some concerns (allocation concealment not described in sufficient detail) | Some concerns (no participant/therapist blinding feasible) | Low | Some concerns (assessor blinding not explicitly described) | Low | Some concerns |

***Notes:*** Domain ratings: "low risk", "some concerns", or "high risk", per Sterne et al., (1). Performance and detection bias domains (D2 and D4) are constrained by the inherent infeasibility of blinding participants/therapists in a perceptible bioelectrical-feedback intervention.

**Supplementary Table S3. Per-domain ROBINS-I ratings for the six non-randomized controlled studies.**

| **Study (Ref.)** | **D1: Confounding** | **D2: Selection of participants** | **D3: Classification of interventions** | **D4: Deviations from intended interventions** | **D5: Missing data** | **D6: Measurement of outcomes** | **D7: Selection of the reported result** | **Overall judgement** |
| --- | --- | --- | --- | --- | --- | --- | --- | --- |
| Doğan-Aslan 2012 (16) | Moderate (limited adjustment for baseline severity) | Moderate | Low | Moderate (no feasible blinding) | Low | Moderate (assessor blinding not described) | Low | Moderate |
| Yang 2024 (17) | Moderate | Moderate | Low | Moderate (no feasible blinding) | Low | Moderate (assessor blinding not described) | Low | Moderate |
| Wang 2023 (18) | Serious (limited information on baseline confounders) | Moderate | Low | Moderate (no feasible blinding) | Moderate (some incomplete outcome reporting) | Moderate (assessor blinding not described) | Low | Serious |
| Xie 2020 (19) | Moderate | Moderate | Low | Moderate (no feasible blinding) | Low | Moderate (assessor blinding not described) | Low | Moderate |
| Bai 2020 (20) | Moderate | Moderate | Low | Moderate (no feasible blinding) | Low | Moderate (assessor blinding not described) | Low | Moderate |
| Hu 2023 (21) | Serious (whole-body vibration co-intervention; limited adjustment for confounders) | Moderate | Low | Moderate (no feasible blinding) | Moderate (some attrition not fully described) | Moderate (assessor blinding not described) | Low | Serious |

***Notes:*** Domain ratings: "low risk", "moderate risk", "serious risk", "critical risk", or "no information", per Sterne et al., (2). Performance bias (D4) is constrained by the inherent infeasibility of blinding participants/therapists in a perceptible bioelectrical-feedback intervention.

**Supplementary Note S3. Complete, executable search strategies for all five databases.**

All searches were executed by the first author (MY) on 31 March 2025, with no language or document-type restrictions other than those described in the eligibility criteria. The same conceptual structure was used in each database, with syntax adapted to that database's field codes and Boolean conventions, as listed below.

**(1) PubMed (NLM). Date executed: 31 March 2025.**

#1 "electromyographic biofeedback"[MeSH Terms]

#2 "EMG biofeedback"[Title/Abstract]

#3 "myoelectric biofeedback"[Title/Abstract]

#4 "bioelectric feedback"[Title/Abstract]

#5 #1 OR #2 OR #3 OR #4

#6 "stroke"[MeSH Terms]

#7 "cerebrovascular accident"[Title/Abstract]

#8 "hemiplegia"[MeSH Terms]

#9 #6 OR #7 OR #8

#10 "motor function"[Title/Abstract]

#11 "rehabilitation"[MeSH Terms]

#12 "hemiplegic"[Title/Abstract]

#13 #10 OR #11 OR #12

#14 #5 AND #9 AND #13

#15 #14 AND ("2010/01/01"[Date - Publication] : "2025/03/31"[Date - Publication])

**(2) Cochrane Library (CENTRAL via Wiley). Date executed: 31 March 2025.**

#1 MeSH descriptor: [Biofeedback, Psychology] explode all trees

#2 ("EMG biofeedback" OR "myoelectric biofeedback" OR "electromyographic biofeedback" OR "bioelectric feedback"):ti,ab,kw

#3 #1 OR #2

#4 MeSH descriptor: [Stroke] explode all trees

#5 MeSH descriptor: [Hemiplegia] explode all trees

#6 ("stroke" OR "cerebrovascular accident" OR "hemiplegia" OR "hemiplegic"):ti,ab,kw

#7 #4 OR #5 OR #6

#8 ("motor function" OR "rehabilitation"):ti,ab,kw

#9 MeSH descriptor: [Rehabilitation] explode all trees

#10 #8 OR #9

#11 #3 AND #7 AND #10

#12 #11 with publication year from 2010 to 2025, in Trials

**(3) EMBASE (Elsevier). Date executed: 31 March 2025.**

#1 'biofeedback'/exp

#2 ('electromyographic biofeedback' OR 'EMG biofeedback' OR 'myoelectric biofeedback' OR 'bioelectric feedback'):ti,ab,kw

#3 #1 OR #2

#4 'stroke'/exp OR 'cerebrovascular accident'/exp OR 'hemiplegia'/exp

#5 ('stroke' OR 'cerebrovascular accident' OR 'hemiplegia' OR 'hemiplegic'):ti,ab,kw

#6 #4 OR #5

#7 'rehabilitation'/exp OR ('motor function' OR 'rehabilitation'):ti,ab,kw

#8 #3 AND #6 AND #7

#9 #8 AND [2010-2025]/py

**(4) Web of Science Core Collection (Clarivate). Date executed: 31 March 2025.**

#1 TS=("electromyographic biofeedback" OR "EMG biofeedback" OR "myoelectric biofeedback" OR "bioelectric feedback")

#2 TS=("stroke" OR "cerebrovascular accident" OR "hemiplegia" OR "hemiplegic")

#3 TS=("motor function" OR "rehabilitation")

#4 #1 AND #2 AND #3

#5 #4 AND PY=(2010-2025), Document Types: (Article OR Review)

**(5) CNKI (China National Knowledge Infrastructure). Date executed: 31 March 2025.**

Search: SU = ('肌电生物反馈' OR 'EMG生物反馈' OR '生物电反馈') AND SU = ('脑卒中' OR '脑梗' OR '脑出血' OR '偏瘫') AND SU = ('康复' OR '运动功能')

Filters: 学科 = 临床医学 / 神经病学 / 康复医学; 时间范围 = 2010-01-01 至 2025-03-31; 文献类型 = 期刊论文.

**References**

1. Sterne JA, Savović J, Page MJ, Elbers RG, Blencowe NS, Boutron I, et al. Rob 2: A Revised Tool for Assessing Risk of Bias in Randomised Trials. *bmj* (2019) 366.

2. Sterne JA, Hernán MA, Reeves BC, Savović J, Berkman ND, Viswanathan M, et al. Robins-I: A Tool for Assessing Risk of Bias in Non-Randomised Studies of Interventions. *bmj* (2016) 355.
